# Effect of diabetes mellitus on short-term prognosis of 227 pyogenic liver abscess patients after hospitalization

**DOI:** 10.1186/s12879-020-4855-9

**Published:** 2020-02-17

**Authors:** Zhaoqing Du, Xingchen Zhou, Junzhou Zhao, Jianbin Bi, Yifan Ren, Jia Zhang, Yuxin Lin, Zheng Wu, Yi Lv, Xufeng Zhang, Rongqian Wu

**Affiliations:** 1 National-Local Joint Engineering Research Center for Precision Surgery & Regenerative Medicine, Shaanxi Provincial Center for Regenerative Medicine and Surgical Engineering, First Affiliated Hospital of Xi’an Jiaotong University, Xi’an, 710061 Shaanxi Province China; 2 Department of Hepatobiliary Surgery, First Affiliated Hospital of Xi’an Jiaotong University, Xi’an, 710061 Shaanxi Province China

**Keywords:** Diabetes mellitus, Pyogenic liver abscess, Prognosis, Survival

## Abstract

**Background:**

Pyogenic liver abscess (PLA) is an inflammatory disease with increasing incidence. When it occurs with diabetes mellitus (DM), the risk of recurrence and mortality may increase. However, the effect of DM on the short-term prognosis of PLA patients after hospitalization remained unknown.

**Methods:**

Two hundred twenty-seven PLA patients who received treatment at the First Affiliated Hospital of Xi’an Jiaotong University from January 2011 to January 2018 were retrospectively enrolled. They were divided into two groups as the DM group (*n* = 61) and the Non-DM group (*n* = 166). In the DM group, HbA1C level < 7% was considered to be good-control of glycaemia (*n* = 23). The clinical characteristics and overall short-term survival were analyzed.

**Results:**

The proportion of PLA patients with DM was 26.87%. In the DM group, there was a higher incidence of hypertension and *Candida spp.* infection. Conservative administration and percutaneous drainage were mainly used in patients with good- (60.87%) and poor-control (60.53%) of glycaemia, respectively. During follow-up, 24 (10.57%) died due to uncontrolled systemic infections and other serious complications. Compared with PLA patients without DM, patients in the DM group had significantly increased 6-month mortality rate after discharge (Log-Rank test, *P* = 0.021). Poor-control of glycaemia did not reduce the six-month survival, while the recurrence rate of PLA within 3 months showed an almost 3-fold increase (13.16% vs. 4.35%). Further multivariate analyses found that DM was the only independent risk factor for the PLA six-month survival (odds ratio [OR]: 3.019, 95% confidence interval [CI]: 1.138–8.010, *P* = 0.026). However, the blood glucose level had no significant effect on the short-term survival of PLA patients with DM (Log-Rank test, *P* = 0.218).

**Conclusions:**

In PLA patients, DM aggravated short-term mortality and blood glucose levels should be well controlled.

## Background

Pyogenic liver abscess (PLA), as a serious infectious disease occurs in the liver parenchyma, has shown a trend of increasing incidence and high mortality recently [1]. Researchers found that PLA had different prevalence in general population over the world, with a ranging from 4 per 10,000 [2] in Europe and Americas to between 18 and 86 per 10,000 [1, 3] in Asia. Because it can cause fatal systemic infections, the mortality rate of PLA within 30 days of hospitalization can be as high as 10% [4]. Especially in the case of combining high risk factors or underlying conditions, serious complications and worse prognosis are more likely [5].

The DM prevalence is relatively high among PLA patients and can be up to 35.3% [4]. Meanwhile, DM status may easier result in severe complications and recurrent infection [6]. In addition, poor-control of blood glucose may aggravate the situation and prognosis [7]. However, the clinical characteristics and short-term survival between PLA patients with and without DM have not been fully investigated before, especially in northwest China. Moreover, research has rarely been published about the effect of blood glycaemic control on these aspects of PLA patients with DM. In this study, we conducted a retrospective study on PLA patients with or without DM, and with good or poor control of blood glucose level, comparing their clinical characteristics and short-term survivals, which might help to improve the diagnosis and prognosis of PLA patients with DM and provide clues of the effect of glycaemic control.

## Methods

### Patients

In this retrospective study, we examined the patients who were diagnosed primarily as PLA in the First Affiliated Hospital of Xi’an Jiaotong University between January 2011 to January 2018. All diagnoses of PLA were based on the clinical features, imaging and laboratory results, blood and *pus* cultures. Details of 422 patients were retrieved through the hospital’s electronic medical record system. Patients with the following conditions were excluded: those who were diagnosed with malignant tumors or other serious cardiovascular diseases, those who were not the first-time suffering from liver abscess, those who had not finished the hospitalization in our center and those who had incomplete medical records or a 180 days follow-up. Finally, 227 patients entered this study (Additional file 1: Table S1). Patients were firstly divided into two groups based on the diagnoses with or without diabetes, and then the patients with diabetes were further divided into two subgroups, good- and poor-control groups of glycaemia. Patients of type II diabetes mellitus all had a clear history of diabetes at admission, and none of them were diagnosed after admission. We defined DM patients with HbA1C level < 7% included in the good-control group and ≥ 7% in the poor-control group. This study was strictly complied with the Treaty of Helsinki and approved by the ethics committee of the First Affiliated Hospital of Xi’an Jiaotong University. Written informed consent were waived as a retrospective research.

### Data collection

All data were collected from the electronic medical records system. The general data we extracted included age, sex, symptoms and signs, underlying conditions, hepatitis status, medicine use, cost and length of hospital stay. Laboratory results at admission were obtained, containing blood routine, liver function, renal function, coagulation, blood and *pus* culture data (involved those who had either and those who had both), plasma glucose, HbA1C level and diabetic vascular diseases.

Diabetic macro-angiopathy collected cardio- and cerebrovascular accidents and lower extremity gangrene, and micro-angiopathy included microvascular lesions of retina and kidney caused by high blood glucose levels. Complications, treatments and outcomes were also gained. Abscess number, diameter, site and gas-forming were measured by ultrasonography and computerized tomography (CT).

### Follow-up

Follow-up for all patients was until June 2018 and lasted for at least 6 months after discharge. The average follow-up time was 820 (IQR: 308, 1261) days. The main contents of the follow-up included the disease status and recurrence of all patients. All patients achieved full follow-up data. The mortality outcome was ascertained from discharge time to the last follow-up. To minimize classification errors, the follow-up was completed by two clinical researchers independently.

### Statistical analysis

For continuous variables, the study was expressed as mean ± standard deviation or median (min-max) whereas categorical variables used frequency and percentage. To calculate the difference between the two groups, we applied the Student’s t-test or Wilcoxon test for continuous variables and the chi-squared test or Fisher’s exact test for categorical data. For three or more groups, we applied the analysis of variance. The factors that found *P* < 0.10 in the univariate analysis were carried forward through the multivariate analysis. Survival curves were calculated by the Kaplan–Meier curve method, and statistical differences were calculated through the Log-Rank test. All statistical analyses were finished by SPSS 23.0 software (IBM Corporation, Armonk, NY, USA). Graphpad prism 8.0 software was used to beautify the survival curves (GraphPad Software, Inc. La Jolla, USA). *P* < 0.05 was considered as statistically significant.

## Results

### Demographic and clinical characteristics

A total of 227 patients with PLA were enrolled in this retrospective study. As shown in Table 1, the average age of total patients was 56 years (range: 11–84 years), and 135 (59.47%) of them were male. Among them, 66 (29.07%) patients had a history of smoking, 43 (18.94%) patients had alcohol drinking history. 43 (18.94%) and 8 (3.52%) patients had hypertension and cirrhosis, respectively. Most patients had a normal (60.79%) or low-grade fever temperature (34.80%) at admission, and the medium time for temperature normalization was 6.39 (range: 0–40). leucocytes abnormally increased in 119 patients (52.42%) on the first day of admission. In this cohort, the number of the abscess was mainly single (75.77%). The maximum diameter of the abscess in 132 patients (58.15%) was between 5 and 10 cm, then followed by a small abscess in 75 cases (33.04%). In 137 (60.35%) patients, the abscess located in the right liver lobe, 34 (14.98%) in the left, and 41 (18.06%) in both. Gas formation was observed in 45 patients (19.82%). In Table 2, 116 blood cultures were collected and only 28 patients (24.14%) were positive. There was no significant difference in the positive proportion between the two groups. Based on *pus* culture from 148 cases, positive growth was found in 98 patients (66.22%). Among the positive patients, 114 patients (85.07%) were Gram-negative bacteria, 18 (13.43%) were Gram-positive bacteria, and 2 (1.49%) were *Candida spp.* infections. *Klebsiella pneumonia* and *Escherichia coli* in the Gram-negative organisms were the two most common bacteria in this study, accounting for 57.46 and 17.91%, respectively. In the Gram-positive organisms, *Streptococcus spp.* (6.72%) was the most pathogens cultured. It was documented that pleural and celiac effusion were the most common complications (18.41, and 7.58%, respectively, Table 3). 136 (59.91%) patients received percutaneous drainage, 28 (12.33%) underwent surgical drainage, and the rest 63 (27.75%) were administrated conservative treatment. 125 (55.07%) patients presented combined antibiotic use. All patients showed outcomes with cured or improved and no patient died during the hospital stay. The average cost of total patients was 4096 dollars.

**Table 1:** Clinical characteristics of the 227 patients with pyogenic liver abscess patients with diabetes or without diabetes presented at admission

Values	Median (range)/*n* (percentage)	Diabetes mellitus	Non-diabetes mellitus	*p* value
n	227	61 (26.87%)	166 (73.13%)	
Age (years)	56 (11–84)	57 (22–84)	56 (11–84)	0.837
Gender (male)	135 (59.47%)	39 (63.93%)	96 (57.83%)	0.406
Underlying conditions				
Smoking	66 (29.07%)	16 (26.23%)	50 (30.12%)	0.567
Drinking	43 (18.94%)	33 (19.88%)	10 (16.39%)	0.552
Hypertension	43 (18.94%)	31 (50.82%)	12 (7.23%)	**< 0.001**
Cirrhosis	8 (3.52%)	2 (3.28%)	6 (3.61%)	0.902
Body temperature at admission (°C)				
35.5 °C~ 37.3 °C	138 (60.79%)	42 (68.85%)	96 (57.83%)	0.319
37.4 °C~ 39 °C	79 (34.80%)	17 (27.87%)	62 (37.35%)	
> 39.1 °C	10 (4.41%)	2 (3.28%)	8 (4.82%)	
Time for temperature normalization (days)	6.39 (0–40)	6.05 (0–24)	6.51 (0–40)	0.593
Laboratory tests				
WBC > 10 × 10^9^/L	119 (52.42%)	23 (37.70%)	96 (57.83%)	**0.007**
WBC < 3.5 × 10^9^/L	4 (1.76%)	3 (4.92%)	1 (0.60%)	0.105
ALT > 40 U/L	108 (47.58%)	25 (40.98%)	83 (50.00%)	0.228
AST > 40 U/L	67 (29.52%)	15 (24.59%)	52 (31.33%)	0.324
ALB < 35 g/L	169 (74.45%)	45 (73.77%)	124 (74.70%)	0.887
TBIL > 17 μmol/L	96 (42.29%)	31 (50.82%)	65 (39.16%)	0.115
PT > 17 s	20 (8.81%)	6 (9.84%)	14 (8.43%)	0.741
APTT > 45 s	26 (11.45%)	7 (11.48%)	19 (11.45%)	0.995
BUN > 7.2 mmol/L	16 (7.27%)	7 (11.67%)	9 (5.63%)	0.213
Cr > 97 μmol/L	30 (13.64%)	7 (11.67%)	23 (14.37%)	0.602
Abscess number				
Solitary abscess	172 (75.77%)	47 (77.05%)	125 (75.30%)	0.785
Multiple abscess	55 (24.23%)	14 (22.95%)	41 (24.70%)	
Maximal diameter of abscess				
≤ 5 cm	75 (33.04%)	24 (39.34%)	51 (30.72%)	0.116
5~10 cm	132 (58.15%)	34 (55.74%)	98 (59.04%)	
> 10 cm	20 (8.81%)	3 (4.92%)	17 (10.24%)	
Abscess site				
Left lobe	34 (14.98%)	6 (9.84%)	28 (16.87%)	0.289
Right lobe	137 (60.35%)	43 (70.49%)	94 (56.63%)	
Both left and right	41 (18.06%)	9 (14.75%)	32 (19.28%)	
Other sites	15 (6.61%)	3 (4.92%)	12 (7.23%)	
Gas forming	45 (19.82%)	15 (24.59%)	30 (18.07%)	0.275
Medicine use				
Steroid hormone	57 (25.11%)	21 (34.43%)	36 (21.69%)	**0.050**
Hepatoprotective drugs	126 (55.51%)	36 (59.02%)	90 (54.22%)	0.519
Immune response enhancer	42 (18.50%)	24 (39.34%)	18 (10.84%)	**< 0.001**

*WBC* white blood cell, *ALT* alanine transaminase, *AST* aspartate transaminase, *ALB* albumin, *TBIL* total bilirubin, *PT* prothrombin time, *APTT* activated partial thromboplastin time, *BUN* blood urea nitrogen, *Cr* creatinine

**Table 2:** Blood and *pus* cultures in pyogenic liver abscess patients with diabetes mellitus (DM) and non-diabetes mellitus (Non-DM)

Values	Blood culture		*Pus* culture	
	DM (*n* = 34)	Non-DM (*n* = 82)	DM (*n* = 34)	Non-DM (*n* = 114)
Positive results	10	18	25	73
Polymicrobial results	1	2	1	4
Gram-negative organisms				
*Klebsiella pneumonia*	3	12	15	47
*Escherichia coli*	4	3	5	12
*Pseudomonas aeruginosa*	1	0	2	1
*Enterobacter cloacae*	0	0	0	2
*Proteus spp.*	0	0	0	2
*Serratia fonticola*	0	1	0	0
*Salmonella enteritidis*	0	0	1	0
*Klebsiella oxytoca*	0	0	0	1
*Bacillus citrate*	0	0	0	1
*Stenotrophomonas maltophilia*	0	0	1	0
Gram-positive organisms				
*Streptococcus spp.*	1	1	0	7
*Enterococcus spp.*	2	0	0	3
*Clostridium spp.*	0	2	0	0
*Staphylococcus spp.*	0	1	0	1
*Candida spp.*	0	0	2	0

**Table 3:** Complications, treatments, outcomes and survival of the pyogenic liver abscess patients with diabetes mellitus and non-diabetes mellitus

Variables	Median (range)/*n* (percentage)	Diabetes mellitus	Non-diabetes mellitus	*p* value
Complications				
Bile leakage	9 (3.96%)	2 (3.28%)	7 (4.22%)	1.000
Intraperitoneal bleeding	10 (4.41%)	3 (4.92%)	7 (4.22%)	0.731
Plumonary infection	5 (2.20%)	2 (3.28%)	3 (1.81%)	0.613
Pleural effusion	51 (18.41)	17 (27.87%)	34 (20.48%)	0.237
Celiac effusion	21 (7.58)	8 (13.11%)	13 (7.83%)	0.208
Treatments				
Percutaneous drainage	136 (59.91%)	29 (47.54%)	107 (64.46%)	**0.024**
Surgical drainage	28 (12.33%)	7 (11.48%)	21 (12.65%)	
Conservative treatment	63 (27.75%)	25 (40.98%)	38 (22.89%)	
Antibiotic use				
Combined	125 (55.07%)	37 (60.66%)	88 (53.01%)	0.305
Single	102 (44.93%)	24 (39.34%)	78 (46.99%)	
Outcomes				
Cured	155 (68.28%)	45 (73.77%)	110 (66.27%)	0.282
Improved	72 (31.72%)	16 (26.23%)	56 (33.73%)	
Death	0 (0%)	0 (0%)	0 (0%)	
Hospital stay (days)	14 (2–52)	15 (3–40)	14 (2–52)	0.444
Total hospitalization expenses (× 1000 dollars)	4.10 (0.39–27.16)	4.24 (0.39–27.16)	4.05 (0.47–18.05)	0.748
Reoccurrence in three months	14 (6.17%)	6 (9.84%)	8 (21.05%)	0.164
Survival/Death in six months	203 (89.43%) /24 (10.57%)	50 (81.97%) /11 (18.03%)	153 (92.17%) /13 (7.83%)	**0.027**

### Associations between diabetes mellitus and patient characteristics

In this study, 227 patients were divided into two groups based on DM. The Non-DM group included 166 (73.13%) patients and the DM group included 61 (26.87%) patients, with an average diabetes duration time of 5.5 years (range: 0.2–30 years). There was no significant difference in gender, age, body temperature at admission, abscess number, size, site, and gas-forming between the two groups (Table 1). However, patients in the DM group exhibited more hypertension but lower leucocytes count (*P* < 0.001, and *P* = 0.007, respectively). Interestingly, the use of steroid hormone (34.43% vs. 21.69%) and immune response enhancer (39.34% vs. 10.84%) was significantly higher in PLA patients with DM than in the non-DM group (*P* < 0.05). Although the infection of Gram-negative and –positive in this study were similar between the two groups, patients in the DM group had higher *Candida spp.* infection than that of in Non-DM group (*P* = 0.052, Table 2). In Table 3, no significant difference had been found in complications, intravenous antibiotics use, outcomes, hospital stay, total costs and reoccurrence in 3 months between the two groups. While the choice of treatments was different between the two groups (*P* = 0.024). Percutaneous drainage was mainly adopted in patients without DM (64.46%), while percutaneous drainage and conservative treatment were mainly performed in patients with DM (47.54 and 40.98%).

### Effect of diabetes mellitus on short-term survival in liver abscess patients

**Fig. 1:**
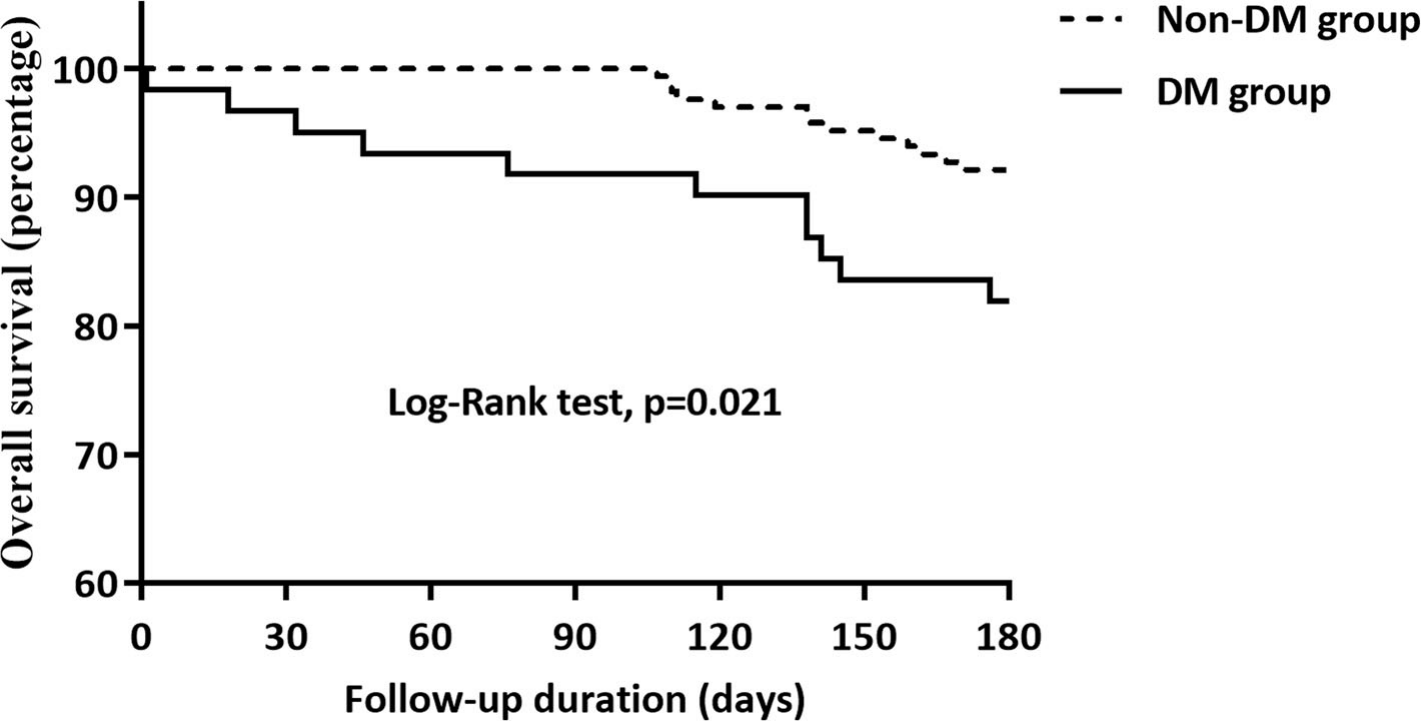
Effect of diabetes mellitus on six-month survival in PLA patients after discharge. Differences in short-term survival rates between PLA patients who combined with diabetes (DM group) and those who did not combine with diabetes (Non-DM group). The survival rate was assessed by the Kaplan-Meier analysis and compared by the Log-Rank test

During the follow-up period, 24 (10.57%) died within 6 months and 14 (6.17%) were diagnosed with PLA recurrence within 3 months after discharge. The PLA patients with DM had lower six-month survival than that of in Non-DM group (81.97% vs. 92.17%, *P* = 0.027) and no significant difference had been found with the reoccurrence rate between the two groups (*P* = 0.164, Table 3). To explore risk factors independently associated with short-term survival after hospitalization in PLA patients, univariable and multivariable analysis were made. Univariate variables with *P* < 0.10 were further analyzed with a multivariate model. As shown in Table 4, diabetes mellitus (OR: 3.019, 95% CI:1.138–8.010, *P* = 0.026) was the only independent risk factor for six-month survival after discharge. Other factors including age > 60 years, gender, cirrhosis, biliary tract infection, abscess number and size, gas forming, *Escherichia coli* infection, and treatment methods were not found to be independent risk factors. The Kaplan-Meier curve of six-month survival after discharge was further estimated concerning DM in Fig. 1. It was found that PLA patients with DM had worse short-term survival than those in the Non-DM group (Log-Rank test, *P* = 0.021).

**Table 4:** Univariate and multivariate analysis of factors associated with PLA six-month survival

Variables	Univariate analysis		Multivariate analysis	
	OR (95%CI)	*P*	OR (95%CI)	*P*
Age > 60 years	2.261 (0.517–9.507)	0.284		
Gender (male)	0.395 (0.092–1.627)	0.212		
Smoking (yes/no)	1.486 (0.345–6.404)	0.595		
Drinking (yes/no)	1.447 (0.282–7.430)	0.658		
Hypertension (yes/no)	1.447 (0.282–7.430)	0.658		
Cirrhosis (yes/no)	4.327 (0.467–40.094)	0.197		
Diabetes (yes/no)	2.589 (1.091–6.144)	**0.031**	3.019 (1.138–8.010)	**0.026**
Biliary tract infection (yes/no)	0.835 (0.099–7.015)	0.868		
Leucocytes > 10 × 10^9^/L	0.533 (0.124–2.284)	0.397		
Hemoglobin < 120 g/L	0.536 (0.106–2.716)	0.451		
Platelet count < 100 × 10^9^/L	1.161 (0.137–9.843)	0.891		
TBIL > 17 μmol/L	2.344 (0.546–10.058)	0.252		
PT > 17 s	3.722 (0.700–19.800)	0.150		
BUN > 7.2 mmol/L	2.19 (0.421–11.394)	0.351		
Cr > 97 μmol/L	1.876 (0.216–16.27)	0.568		
Abscess number	0.382 (0.046–3.175)	0.373		
Diameter of abscess	1.029 (0.337–3.141)	0.960		
Abscess site	1.822 (0.905–3.667)	0.093	2.156 (0.832–5.586)	0.114
Gas-forming	5.024 (0.531–47.518)	0.159		
*Klebsiella pneumonia* infection	0.315 (0.036–2.758)	0.297		
*Escherichia coli* infection	2.194 (0.904–5.325)	0.083	0.226 (0.038–1.339)	0.101
Treatments	1.046 (0.327–3.344)	0.940		

*TBIL* total bilirubin, *PT* prothrombin time, *BUN* blood urea nitrogen, *Cr* creatinine

### Subgroups analysis in PLA patients with DM

**Fig. 2:**
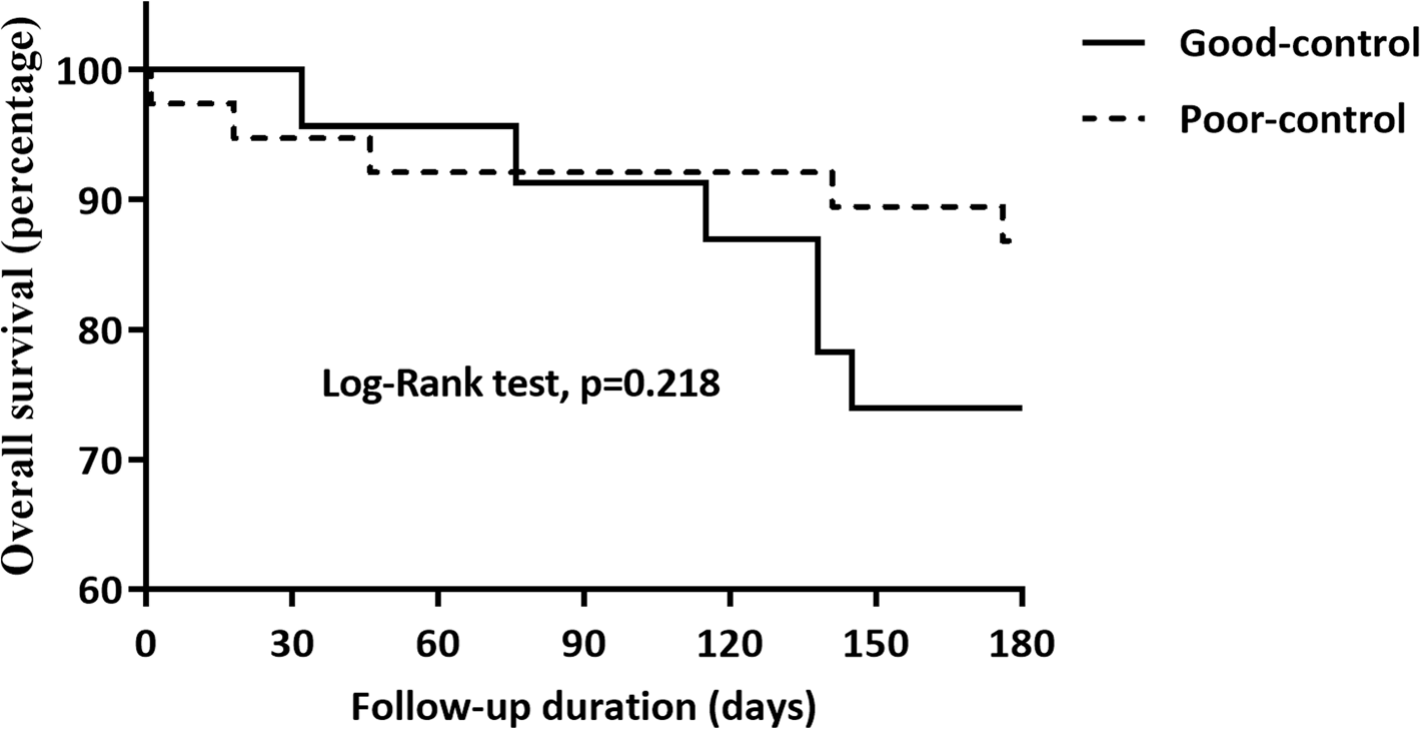
Effect of controlled blood glycaemia level on six-month survival in PLA patients with diabetes after discharge. Differences in short-term survival rates between PLA patients with diabetes who performed with good-control of glycaemia (Good-control group) and those who did not (Poor-control group). The survival rate was assessed by the Kaplan-Meier analysis and compared by the Log-Rank test

Abnormal glycaemia level in the DM group was considered as a potent risk factor of short-term survival in PLA patients, which was further investigated in subgroups. All the patients were classified into two subgroups according to the HbA1C level. The clinical characteristics, laboratory results, abscess information, treatments and outcomes were compared between the two subgroups in Table 5. No difference was presented between the two subgroups in age, gender, diabetes duration time, underlying conditions, diabetic vascular diseases, laboratory tests, abscess characteristics, medicine use, outcomes, and hospital stay, etc. However, there was an obvious difference in the selection of treatments between the two subgroups (*P* = 0.027). Percutaneous drainage was mainly performed in patients with poor-control of glycaemia (60.53%) and conservative treatment was mainly adopted in patients with good-control of glycaemia (60.87%). Besides, combined intravenous antibiotics showed a higher proportion in poor-control group (71.05%, *P* = 0.033). Although there was no significant difference, total hospitalization costs and relapsed PLA within 3 months after discharge showed an obvious increase in the poor-control group compared to good-control group (6037 vs. 3154 dollars, 13.16% vs. 4.35%, respectively). Kaplan-Meier curve with different glycaemia levels in terms of six-month survival was shown in Fig. 2. No difference was unfolded between the two groups in Kaplan-Meier plots (Log-Rank test, *P* = 0.218).

**Table 5:** The clinical characteristics of pyogenic liver abscess with DM between poor-control of glycaemia group and good-control of glycaemia group

Variables	good-control of glycaemia (*n* = 23)	poor-control of glycaemia (*n* = 38)	*p* value
Age (years)	57 (22–84)	58 (23–80)	0.951
Gender (male)	15 (65.22%)	24 (63.16%)	0.871
Diabetes duration (years)	5.4 (0.4–27)	5.5 (0.2–30)	0.952
Underlying condition			
Smoking	8 (34.78%)	8 (21.05%)	0.237
Drinking	5 (21.74%)	5 (13.16%)	0.603
Hypertension	6 (27.27%)	6 (15.79%)	0.461
Diabetic vascular diseases			
Micro-angiopathy	2 (8.70%)	8 (21.05%)	0.294
Macro-angiopathy	2 (8.70%)	3 (7.89%)	1.000
Laboratory tests			
WBC > 10 × 10^9^/L	7 (30.43%)	16 (42.11%)	0.362
WBC < 3.5 × 10^9^/L	2 (8.70%)	1 (2.63%)	0.652
ALT > 40 U/L	12 (52.17%)	13 (34.21%)	0.167
AST > 40 U/L	7 (30.43%)	8 (21.05%)	0.410
ALB < 35 g/L	16 (69.57%)	29 (76.32%)	0.561
TBIL > 17 μmol/L	14 (60.87%)	17 (44.74%)	0.222
PT > 17 s	4 (17.39%)	2 (5.26%)	0.272
APTT > 45 s	3 (13.04%)	4 (10.53%)	1.000
BUN > 7.2 mmol/L	2 (8.70%)	5 (13.51%)	0.697
Cr > 97 μmol/L	2 (8.70%)	5 (13.51%)	0.697
Abscess number			
Solitary abscess	18 (78.26%)	29 (76.32%)	0.861
Multiple abscess	5 (21.74%)	9 (23.68%)	
Maximal diameter of abscess			
≤ 5 cm	11 (47.83%)	13 (34.21%)	0.178
5~10 cm	11 (47.83%)	23 (60.53%)	
> 10 cm	1 (4.35%)	2 (5.26%)	
Abscess site			
Left lobe	4 (17.39%)	2 (5.36%)	0.220
Right lobe	13 (56.52%)	30 (78.95%)	
Both left and right	5 (21.74%)	4 (10.53%)	
Other sites	1 (4.35%)	2 (5.26%)	
Medicine use			
Steroid hormone	10 (43.48%)	11 (28.95%)	0.247
Hepatoprotective drugs	14 (60.87%)	22 (57.89%)	0.819
Immune response enhancer	10 (43.48%)	14 (36.84%)	0.607
*Klebsiella pneumonia* infection	4 (17.39%)	11 (28.95%)	0.310
*Escherichia coli* infection	2 (8.70%)	3 (7.89%)	1.000
Treatments			
Percutaneous drainage	6 (26.09%)	23 (60.53%)	**0.027**
Surgical drainage	3 (13.04%)	4 (10.53%)	
Conservative treatment	14 (60.87%)	11 (28.95%)	
Antibiotic use			
Combined	10 (43.48%)	27 (71.05%)	**0.033**
Single	13 (56.52%)	11 (28.95%)	
Outcomes			
Cured	16 (69.57%)	29 (76.34%)	0.565
Improved	7 (30.43%)	9 (23.68%)	
Death	0 (0%)	0 (0%)	
Hospital stay (days)	15 (3–40)	15 (3–28)	0.986
Total Hospitalization expenses (× 1000 dollars)	2.21 (3.15–7.79)	6.04 (0.61–27.16)	0.056
Reoccurrence in three months	1 (4.35%)	5 (13.16%)	0.395
Survival (yes/no) in six months	17 (73.91%)	33 (86.84%)	0.353

*WBC* white blood cell, *ALT* alanine transaminase, *AST* aspartate transaminase, *ALB* albumin, *TBIL* total bilirubin, *PT* prothrombin time, *APTT* activated partial thromboplastin time, *BUN* blood urea nitrogen, *Cr* creatinine

## Discussion

The increasing incidence and high mortality of PLA patients with diabetes mellitus has become an important health problem in the hepatobiliary system that plagues humans [6]. As a risk factor for PLA, DM can directly lead to liver damage, abnormal bile secretion, and increasing portal vein infection [8–10]. DM can also cause systemic metabolic disorders and impaired immunity, which weakens the ability of the liver to clear bacteria, making the bacteria easy to colonize and multiply to form abscesses [11]. Studies have confirmed that although the incidence of DM in PLA patients is regionally different in Asia, Europe, and America, it still can be as high as 23 to 44.9% [7, 12–14]. Even in PLA patients caused by *Klebsiella pneumonia*, the incidence of DM is higher as 49.7%. Unexpectedly, this number is likely to grow in recent years with the increasing PLA [14]. In this study, the ratio reached 26.87%.

PLA and DM are high wasting diseases and often progress rapidly when both occur. Meanwhile, the development of the PLA has unique characteristics, and the mortality rate is high during the onset period [15]. Combined with DM in PLA patients should have a significant impact on overall survival, especially in the short onset period. Researchers believe that the physique of DM patients tends to be fragile when suffered from PLA, the condition will be complicated and the mortality will be higher [16]. They discovered that PLA patients with and without DM had a mortality rate of 24.8 and 18.0% within 30 days after discharge, respectively [16]. However, other studies agreed that DM was not an independent risk factor for prognosis in PLA patients [3, 6]. In this study, the six-month follow-up was performed on 227 PLA patients who were retrospectively enrolled. We found that the overall mortality rate was 10.57% within 6 months after discharge, which was slightly lower than similar studies [15]. Moreover, DM existed as the only independent risk factor for six-month survival in PLA patients after hospitalization in this cohort and other high-risk factors, such as age > 60 years, cirrhosis, diameter of the abscess, and gas-forming were not statistically significant in the multivariate model [15, 17]. Compared with 7.83% in PLA patients without DM, the mortality dramatically increased to 18.03% in patients with DM. However, in the further subgroups, poor-control of glycaemia didn’t reduce the short-term survival, but the recurrence rate of PLA within 3 months after discharge showed a 3-fold increase (13.16% vs. 4.35%). It can be interpreted as poor-control of glycaemia inducing a higher infection and more abscess numbers by a recent similar study [7].

In PLA patients with DM, blood pressure was significantly high, which suggested that these cases had vascular diseases [18]. Although there was no statistical difference in diabetic vascular diseases between the two subgroups, the incidence of micro-angiopathy in the poor-control group (21.05%) was significantly higher than in the good-control group (8.70%), which might be due to the metabolic disorders in DM patients. Unexpectedly, leukocytes count showed a higher abnormal at admission in the Non-DM group, which might be related to the heavier inflammatory status in this group. Bacterial culture revealed that *Klebsiella pneumonia* and *Escherichia coli* were still the two most common infectious pathogens, whether or not combined with DM [6, 12, 19]. Interestingly, *pus* culture results illustrated 2 cases of *Candida spp.* infection in the DM group. *Candida spp.* is rare in PLA, but it is highly related to mortality [20]. The infection rate of *candida spp.* in this study was only 0.88% (2/227), but all occurred in the DM group, probably due to the vulnerable immune system in these cases. Besides, DM patients may be more common in gas-forming [21], but the slightly higher rate has been found in this study (24.59% vs. 18.07%).

The administration of PLA with DM is commonly more complex than simple PLA. Hyperglycemia in tissue easily causes cell hyperosmotic, regeneration and repair function weakening, which then may delay puncture healing [22]. In our study, PLA patients without DM were mainly operated with percutaneous drainage, while in DM group, percutaneous drainage and simple conservative management were mainly used. Interestingly, in further subgroups, patients with poor blood glycemic control were mainly performed with percutaneous drainage, while patients with good control were mostly treated with the conservative method. It seems that the diameter of abscesses in the poor-controlled group is generally larger in this study [23]. Meanwhile, the use of steroid hormone and immune response enhancers were significantly higher in the DM group, which may be related to the decreased and vulnerable immune system in DM patients. In the poor-control of glycaemia group, PLA patients with a maximum diameter of abscess ≥5 cm accounted for 65.79%, while those in good-control group were only 52.17% [24]. It was notable that the poor-controlled group had a higher proportion of combined antibiotic administration and the good-controlled group presented with a single antibiotic [7]. In addition, the hospitalization cost in the poor-controlled group was twice that of the good-controlled group, although there was no significant difference in hospital stay between the two groups [25]. Previous studies have shown that gas-forming nature and higher creatinine are risk predictors of fatality for PLA patients [6]. However, in our multivariate model, they were not illustrated to be independent risk factors affecting six-month survival after hospitalization.

The limitations of this study are mainly focused on its retrospective and single-centered traits, while because of its large sample size, the conclusions we have gained can still be used as a helpful reference for other researchers and clinicians, especially further enriching the experience of diagnosis and treatment of PLA patients with DM in northwestern China. Moreover, as the follow-up time in the present study was only 6 months, further longer follow-up and expanded sample size will be needed to comment on the long-term administrations and outcomes of PLA patients with DM.

## Conclusions

Underlying with DM is common in PLA patients, whose epidemiology and administrations show unique specificities. PLA patients with DM have a higher incidence of hypertension and *Candida spp.* infection. In the DM group, patients were mainly treated with the conservative method and more combined antibiotics use. During the follow-up period, the DM group showed a higher mortality rate than that in the Non-DM group. It was found that DM aggravated six-month mortality after hospitalization and presented to be the only independent risk factor for the short-term survival in PLA patients. Moreover, poor-control of glycaemia level might cause different treatments and relapse PLA more frequently. Hence, DM is an important risk factor for PLA patients and effective management of blood glucose levels should be recommended.

## Supplementary information

**Additional file 1:** **Table S1.** Flowchart for selection of 227 PLA patients in this study.

## Data Availability

All data used or analyzed in this study are included in this published article.

## Abbreviations

ALB: Albumin
ALT: Alanine aminotransferase
APTT: Activated partial thromboplastin time
AST: Aspartate aminotransferase
BUN: Blood urea nitrogen
Cr: Creatinine
DM: Diabetes mellitus
Non-DM: Non-diabetes Mellitus
PLA: Pyogenic liver abscess
PT: Prothrombin time
TBIL: Total bilirubin
WBC: White blood cell
