# Supplementary material for: Effect of diabetes mellitus on short-term prognosis of 227 pyogenic liver abscess patients after hospitalization
Source: BMC Infect Dis. 2020 Feb 17;20:145. doi: 10.1186/s12879-020-4855-9 (PMC7027105; doi:10.1186/s12879-020-4855-9)
Supplement: Supplementary file 1 — Additional file 1: Table S1. Flowchart for selection of 227 PLA patients in this study. [file 12879_2020_4855_MOESM1_ESM.docx]

**Supplementary information**

**Effect of Diabetes Mellitus on short-term prognosis of 227 pyogenic liver abscess patients after hospitalization**

Zhaoqing Du, Xingchen Zhou, Junzhou Zhao, Jianbin Bi, Yifan Ren, Jia Zhang, Yuxin Lin, Zheng Wu, Yi Lv, Xufeng Zhang, Rongqian Wu

**Table S1.** Flowchart for selection of 227 PLA patients in this study.

|  | | Total PLA patients (n=422) | | *P* value |
| --- | --- | --- | --- | --- |
|  |  | PLA patients with DM (n=137) | PLA patients without DM (n=285) |  |
| Exclusion criteria | Incomplete follow-up | 23 (16.79%) | 45 (15.79%) | 0.794 |
|  | Incomplete medical records | 15 (10.95%) | 28 (9.82%) | 0.721 |
|  | Malignant tumors | 17 (12.41%) | 10 (3.51%) | **<0.001** |
|  | Serious cardiovascular | 4 (2.92%) | 10 (3.51%) | 0.749 |
|  | Non-first occurrence of PLA at admission | 17 (12.41%) | 26 (9.12%) | 0.296 |
| Enrolled patients in the following study | | 61 (44.53%) | 166 (58.25%) |  |
